# Electrochemical nanostructured CuBTC/FeBTC MOF composite sensor for enrofloxacin detection

**DOI:** 10.3762/bjnano.15.120

**Published:** 2024-11-28

**Authors:** Thi Kim Ngan Nguyen, Tien Dat Doan, Huy Hieu Luu, Hoang Anh Nguyen, Thi Thu Ha Vu, Quang Hai Tran, Ha Tran Nguyen, Thanh Binh Dang, Thi Hai Yen Pham, Mai Ha Hoang

**Affiliations:** 1 Graduate University of Science and Technology, Viet Nam Academy of Science and Technology, Ha Noi, Vietnam; 2 Faculty of Chemistry, TNU-University of Sciences, Tan Thinh Ward, Thai Nguyen City, Vietnam; 3 Institute of Chemistry, Vietnam Academy of Science and Technology, 18 Hoang Quoc Viet Street, Cau Giay District, Ha Noi, Vietnam; 4 Hanoi University of Industry, 298 Cau Dien Street, Bac Tu Liem District, Ha Noi, Vietnam; 5 National Key Laboratory of Polymer and Composite Materials, Ho Chi Minh City University of Technology, Vietnam National University, Ho Chi Minh City, 70000, Vietnam; 6 Center for High Technology Research and Development, Vietnam Academy of Science and Technology, 18 Hoang Quoc Viet Street, Cau Giay District, Ha Noi, Vietnam

**Keywords:** CuBTC, electrochemical sensor, enrofloxacin, FeBTC, metal-organic framework

## Abstract

A novel electrochemical sensor for the detection of enrofloxacin (ENR) in aqueous solutions has been developed using a carbon paste electrode modified with a mixture of metal-organic frameworks (MOFs) of CuBTC and FeBTC. These MOFs were successfully synthesized via a solvothermal method and characterized using various techniques, including X-ray diffraction, Fourier-transform infrared spectroscopy, Brunauer–Emmett–Teller analysis, and X-ray photoelectron spectroscopy. The MOF mixture exhibited a particle size ranging from 40 to 100 nm, a high surface area of 1147 m^2^/g, a pore volume of 0.544 cm^3^/g, and a capillary diameter of 1.50 nm. Additionally, energy-dispersive X-ray mapping demonstrated the uniform distribution of the two MOFs within the electrode composition. The synergistic effect of the electrocatalytic properties of CuBTC and the high conductivity of FeBTC significantly enhanced the electrochemical response of ENR, increasing the signal by more than ten times compared to the unmodified electrode. Under optimal analytical conditions, the sensor exhibited three dynamic ranges for ENR detection, that is, 0.005 to 0.100 µM, 0.1 to 1.0 µM, and 1 to 13 µM, with coefficients of determination of 0.9990, 0.9954, and 0.9992, respectively, depending on the accumulation duration. The sensor achieved a low detection limit of 3 nM and demonstrated good reproducibility, with a relative standard deviation of 3.83%. Furthermore, the sensor demonstrated effective performance in analysing tap and lake water samples, with recovery rates ranging from 90.2% to 121.3%.

## Introduction

Enrofloxacin (ENR) is a quinolinemonocarboxylic acid and a third-generation fluoroquinolone. This antibiotic is extensively used in livestock and aquaculture as an antibacterial agent, showing high activity against both Gram-negative and Gram-positive bacteria [1–2]. ENR, like other fluoroquinolones, is used to treat susceptible bacteria responsible for infections of the skin and soft tissue. The overuse of ENR causes severe adverse effects, including skeletal, reproductive, immune, and digestive disorders; moreover, ENR cannot be completely absorbed by most animals, it easily enters the environment through the excrement of organisms, also in the form of metabolites [3–4]. Nowadays, the concentration of ENR in products, especially animal products intended for human consumption is limited in the USA, the EU, and China [1,4]; the FDA also bans the use of ENR in both poultry and livestock [5]. Therefore, the determination of ENR residues is an urgent requirement. Various analytical techniques and methods have been developed to detect ENR, including microbiological methods, such as enzyme-linked immunosorbent assay (ELISA) [6], chromatography-mass spectrometry (LC-MS), chromatography (HPLC, UPLC), [3,7–8], flow immunochromatographic assays [9], and fluorescence measurements [10]. However, these methods have high requirements regarding instruments, chemicals, time consumption, and preparation of the samples. Electrochemical methods to detect ENR have attracted great attention because of benefits including high accuracy, simplicity, and environmental friendliness [11–14]. Electrochemical methods exhibited high efficacy in the detection of ENR with quick response, low cost, and simple handling; hence, these methods are promising to quantify ENR [15–16].

Noble metals and metal-organic frameworks (MOFs) are the most typical electrode materials to detect antibiotics [17–18]. MOFs emerged as the outstanding material for electrochemical sensor applications because of their high loading quantity and surface area, defined structures, and chemical stability [19–20]. Since the first report by Yaghi and his group in 1994 [21], MOFs have attracted great attention. The first publication related to a ﬂuorescent sensor was presented by You et al. in 2002 [22]. MOFs are crystalline hybrid materials with network structures formed by the self-assembly of metal ions or metal clusters and organic ligands, which give them ultrahigh porosity and enormous internal surface area. However, using MOFs for electrochemical sensors has some limitations associated with the low conductivity of MOFs. Therefore, the coupling with conducting materials, such as carbon-based materials, metal nanoparticles, and polymers, has been performed to enhance the electron charge transfer of MOFs [23–24]; single MOFs combined with carbon-based materials have been extensively studied [12,25–28]. A sensitive and simplified electrochemical sensor using a Cu_3_(BTC)_2_-modified carbon paste electrode for detecting 2,4-dichlorophenol was reported by Dong and his group [29]. Owing to the large speciﬁc surface area, high absorption capacity, and an acceptable eﬃcacy of the electron transfer, Cu_3_(BTC)_2_ exhibited a good sensitivity to 2,4-dichlorophenol in the range from 0.04 to 1.00 μM with a limit of detection (LOD) of 9 nM in diﬀerential pulse voltammetry measurements. Moreover, the combination of metal oxides and MOFs showed better electrochemical detection ability than pristine MOFs. For example, Wang et al. developed a MOF/TiO_2_ composite to quantify chlorogenic acid in a range from 0.01 to 1.00 μM with a low LOD of 7 nM [30]. Utilizing carbon-based materials can provide not only enhanced electron transfer but also catalytic functions for the MOFs. A sensor for simultaneously detecting hydroquinone and catechol in water using a Cu-MOF–graphene composite was developed by Li and colleagues. Measurement ranges beginning at 1.0 × 10^−6^ and 1.0 × 10^−3^ M were achieved, with LODs of 5.9 × 10^−7^ M and 3.3 × 10^−7^ M for hydroquinone and catechol, respectively [31]. In addition, the coupling of MOFs with conducting polymers was investigated to modulate their electrical properties. Conducting poly(3,4-ethylenedioxythiophene) nanotubes were coated with porphyrin-based MOFs to detect dopamine in the range of 2 × 10^−6^ to 270 × 10^−6^ M with a LOD of 0.04 × 10^−6^ M [32]. The combination of two types of MOFs has been rarely investigated for the detection of antibiotics.

To our current knowledge, no reports have been made on the use of electrodes modified with a mixture of MOFs for the detection of enrofloxacin. In this study, a sensor was developed using a mixture of CuBTC and FeBTC to modify carbon paste electrodes for the determination of enrofloxacin. The characterization and properties of the fabricated electrode, including molecular structure, morphology, and electrochemical characteristics, were thoroughly investigated using various analytical techniques. The synergistic effects of CuBTC and FeBTC are considered promising for enhancing the performance of enrofloxacin sensors in aqueous solutions.

## Results and Discussion

### Characterization of (Cu)(Fe)BTC

**Figure 1:**
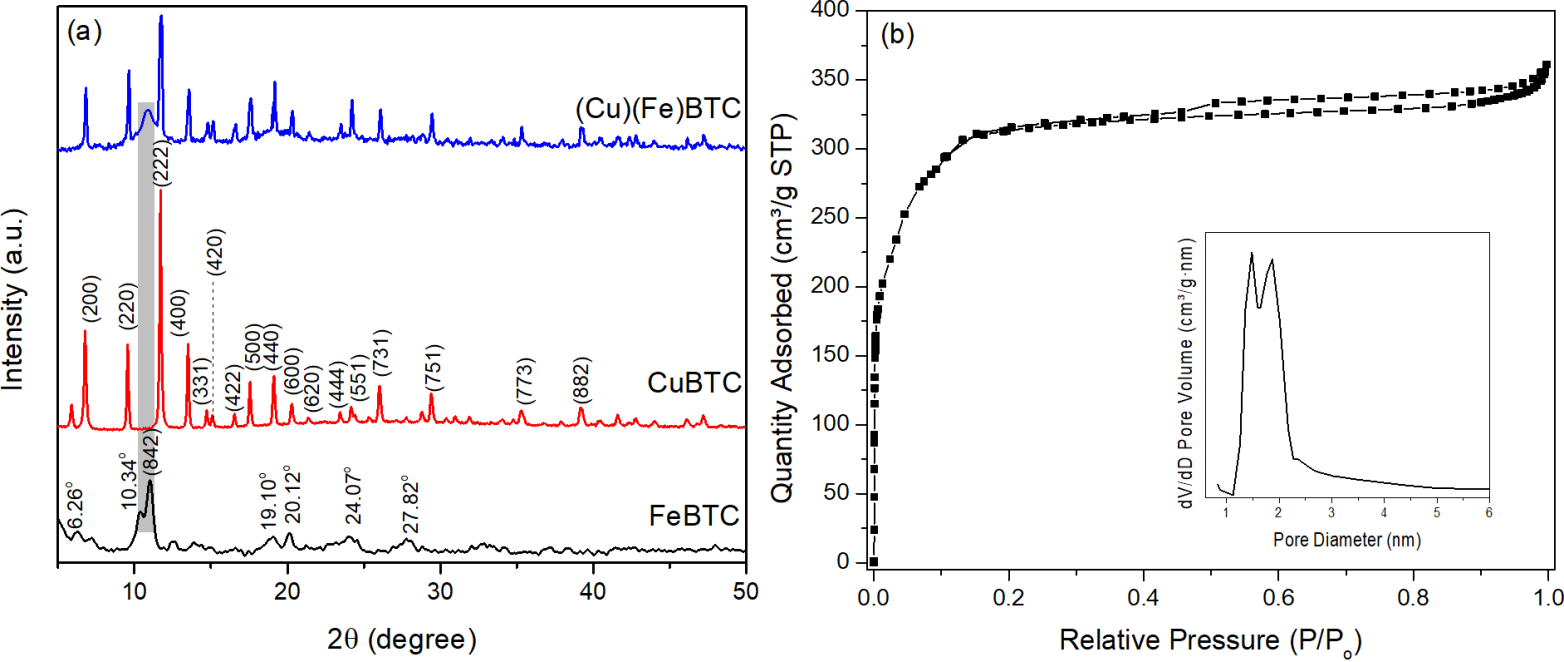
(a) XRD pattern and (b) N_2_ adsorption/desorption isotherms of the (Cu)(Fe)BTC sample.

In the XRD pattern in Figure 1a, the FeBTC sample shows peaks at 2θ values of 6.26°, 10.34°, 10.85°, 19.10°, 20.12°, 24.07°, and 27.82°, which are associated with the crystalline structure of FeBTC (CCDC card No. 640536). The CuBTC sample exhibits reflection peaks at 2θ values of 6.77° (200), 9.65° (220), 11.73° (222), 13.57° (400), 14.76° (331), 15.24° (420), 16.6° (422), 17.56° (500), 19.16° (440), 20.36° (600), 21.40° (620), 23.55° (444), 24.27° (551), 26.11° (731), 29.47° (751), 35.30° (773), and 39.21° (882), characteristic for the CuBTC phase (CCDC card No. 112954) [28,33–34]. The XRD pattern of (Cu)(Fe)BTC exhibits a characteristic peak at 2θ of 10.85° assigned to the (842) crystal planes of the Fe-BTC phase, and all characteristic peaks of CuBTC appear. It can be observed that the characteristic peaks of pure FeBTC have a much lower intensity than those of pure CuBTC; therefore, the intensity of the characteristic peaks of the CuBTC phase predominates in the (Cu)(Fe)BTC pattern. The simultaneous presence of FeBTC and CuBTC phases in the (Cu)(Fe)BTC sample indicates the successful synthesis of (Cu)(Fe)BTC composite.

The N_2_ adsorption/desorption isotherms of the (Cu)(Fe)BTC sample are of type I with H4 hysteresis ring according to IUPAC classification (Figure 1b) [35]. The N_2_ adsorption isotherm for (Cu)(Fe)BTC shows rapid adsorption of nitrogen in the low-pressure range (*P*/*P*_0_ < 0.1), suggesting a predominance of micropores [36]. Surface area, pore volume, and capillary diameters of the (Cu)(Fe)BTC sample are 1147 m^2^/g, 0.544 cm^3^/g, and 1.50 and 1.90 nm, respectively. Total pore volume, average pore diameter, and surface area of FeBTC are 1.46 cm^3^/g, 2.38 nm, and 1211 m^2^/g, respectively [37]. Surface area, total pore volume, and average pore diameter of CuBTC are 1134 m^2^/g, 0.49 cm^3^/g, and 1.74 nm, respectively [38].

**Figure 2:**
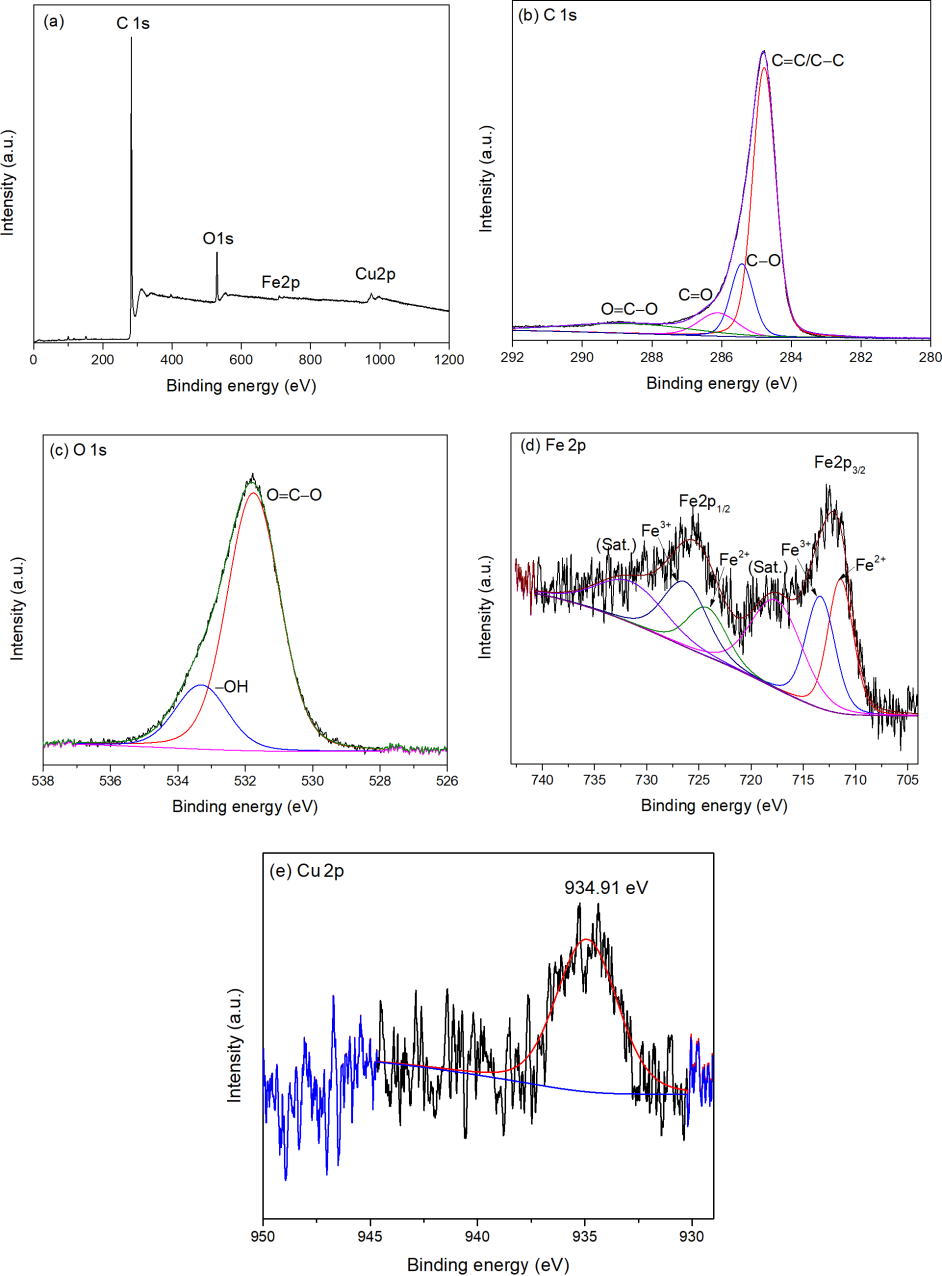
Full-scan (a) and high-resolution C 1s (b), O 1s (c), Fe 2p (d), and Cu 2p (e) XPS spectra of the (Cu)(Fe)BTC sample.

The full-scan XPS spectrum of (Cu)(Fe)BTC (Figure 2a) shows the existence of C (284 eV), O (532 eV), Fe (712 and 726 eV), and Cu (935 eV). The C 1s XPS spectrum of the (Cu)(Fe)BTC sample (Figure 2b) reveals four peaks attributed to C=C/C–C (284.78 eV), C–O (285.42 eV), C=O (286.11 eV), and O–C=O (288.62 eV) [39]. The high-resolution O 1s XPS spectrum (Figure 2c) can be decomposed into oxygen in carboxyl groups (531.74 eV) and physically adsorbed –OH groups (533.30 eV) [40].

In the Fe 2p XPS spectra (Figure 2d), the binding energy peaks of Fe 2p_3/2_ correspond to Fe^2+^ and Fe^3+^ with peak positions at 711.42 and 713.35 eV, respectively. The Fe 2p_1/2_ peaks correspond to Fe^2+^ and Fe^3+^ with central peak positions at 724.20 and 726.31 eV, respectively. In addition, there are two shake-up satellites at 718.08 and 732.04 eV (denoted as sat.). The Fe^2+^ and Fe^3+^ components account for 51.62% and 48.38% in the (Cu)(Fe)BTC determined by peak area. In the Cu 2p XPS spectrum (Figure 2e), the binding energy peak of Cu 2p_3/2_ (934.9 eV) can be attributed to Cu^2+^.

### Characterization of (Cu)(Fe)BTC@CPE

#### Morphology

**Figure 3:**
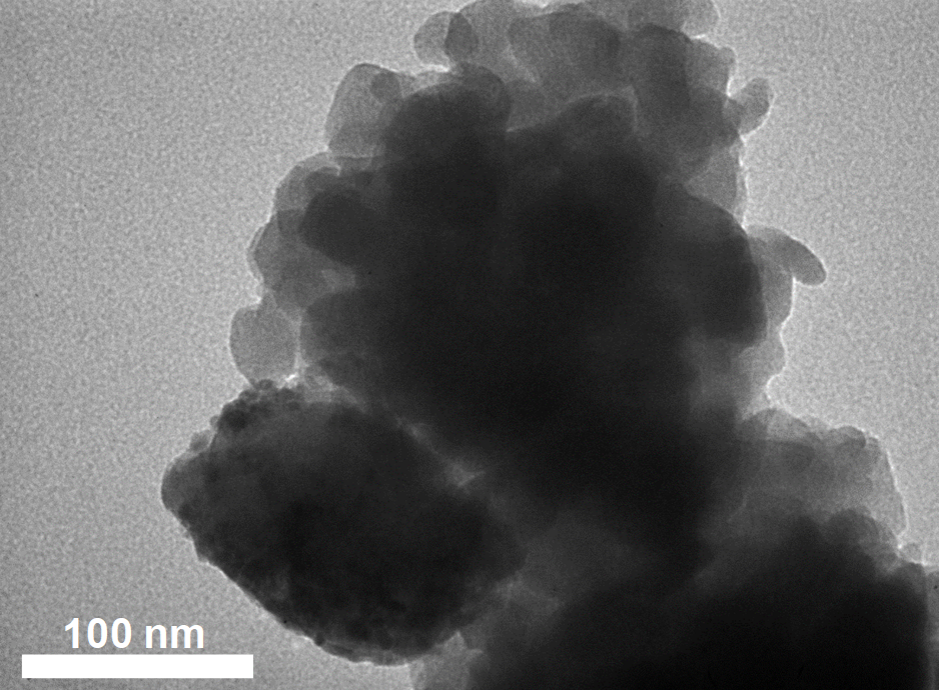
TEM image of (Cu)(Fe)BTC sample.

The TEM image of the (Cu)(Fe)BTC sample shows unevenly distributed particles with different particle sizes fluctuating in the range of 40 to 100 nm (Figure 3).

**Figure 4:**
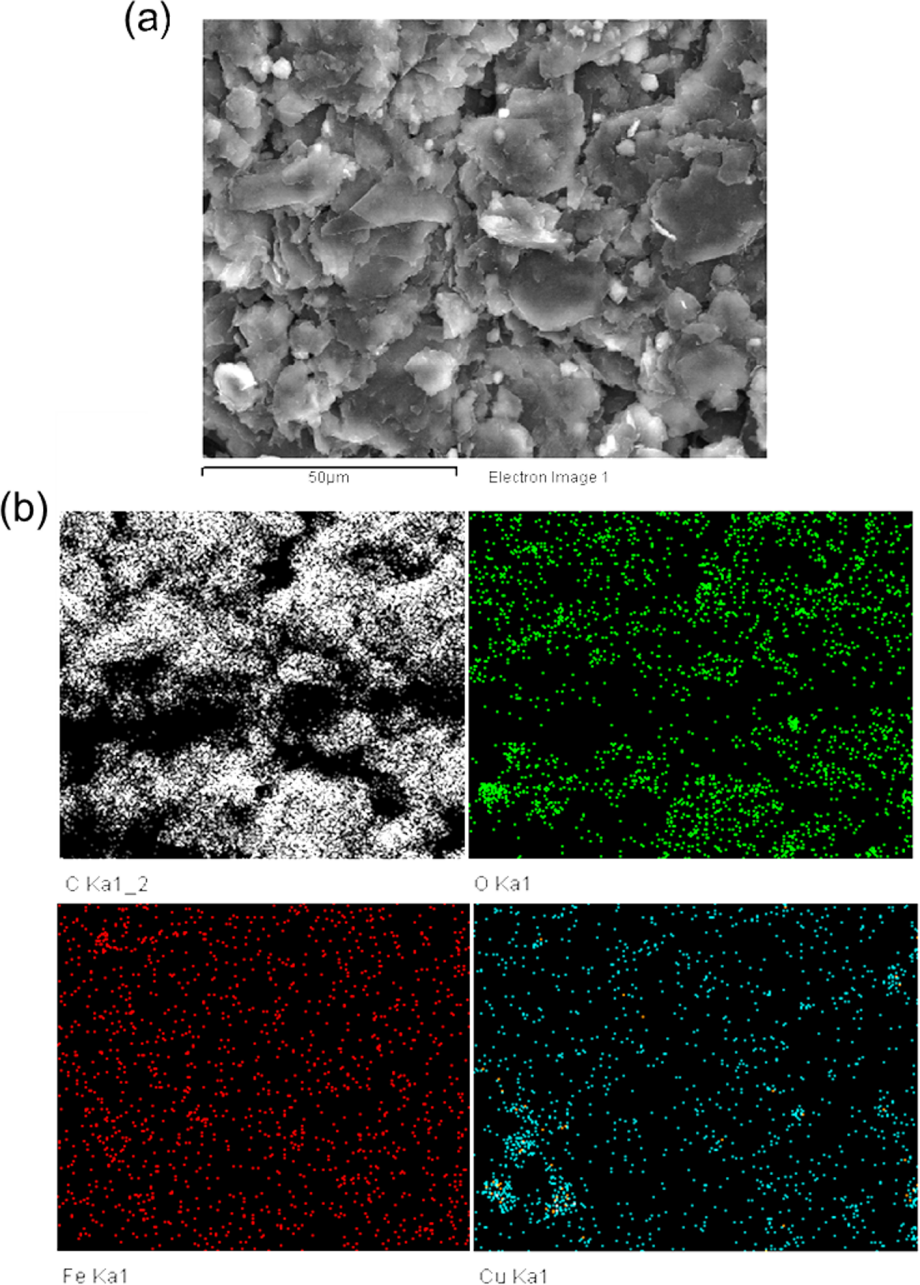
SEM images of (Cu)(Fe)BTC@CPE (a) and EDX maps of (Cu)(Fe)BTC@CPE (b).

After mixing the electrode blend, SEM and EDX mapping were used to investigate the distribution of CuBTC and FeBTC on the electrode surface (Figure 4). Figure 4a clearly shows the scattering of bright CuBTC and FeBTC particles on the graphite flakes. Figure 4b confirms the homogenous distribution of Fe, Cu, and O in the electrode material.

#### Electrochemical property

**Figure 5:**
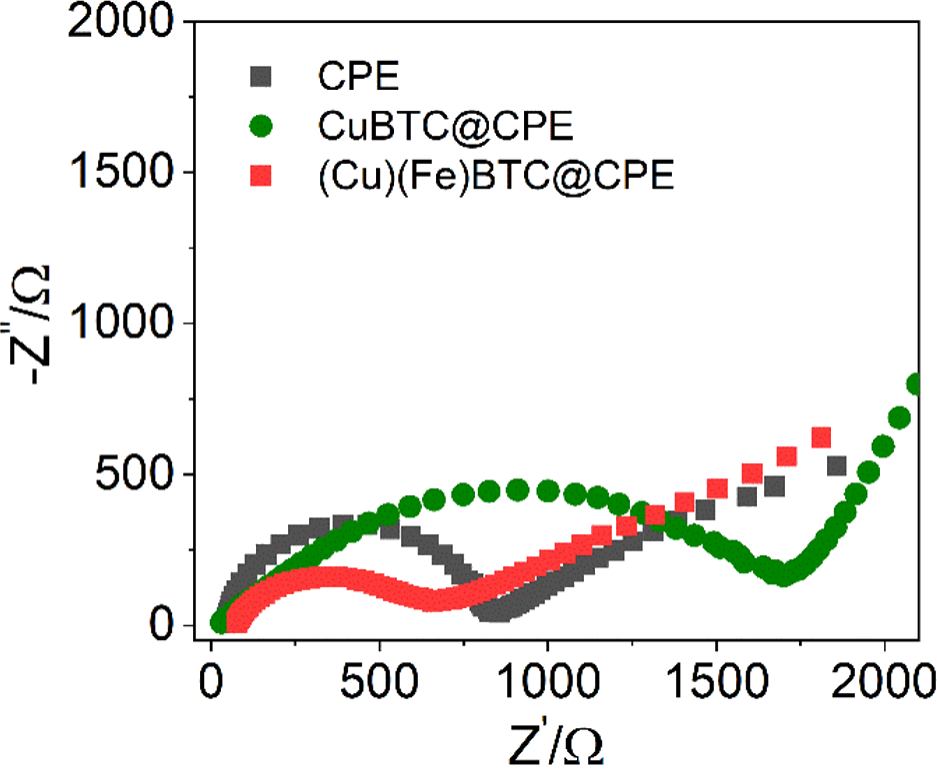
Nyquist plots of the modified electrodes in 0.1 M KCl solution containing 5 mM [Fe(CN)_6_]^3−/4−^.

The Nyquist diagrams in Figure 5 were obtained using electrochemical impedance spectroscopy (EIS) in a 0.1 M KCl solution containing 5 mM [Fe(CN)_6_]^3−/4−^. Based on the EIS analysis, the charge transfer resistances (*R*_ct_) of CPE, CuBTC@CPE, and (Cu)(Fe)BTC@CPE were calculated to be 742, 1710, and 547 Ω, respectively. It is evident that CuBTC increased the *R*_ct_ of the CuBTC@CPE electrode more than twofold compared to the CPE electrode, likely because of its low electron conductivity [41–42]. However, the addition of FeBTC, which has a low *R*_ct_ of 220 Ω and significantly better conductivity [37], to the (Cu)(Fe)BTC@CPE led to reduction to about a third in *R*_ct_. This reduction in charge transfer resistance is advantageous for the enhancement of electrochemical oxidation on the electrode surface.

### Electrochemical behaviour of enrofloxacin on the (Cu)(Fe)BTC@CPE electrode

**Figure 6:**
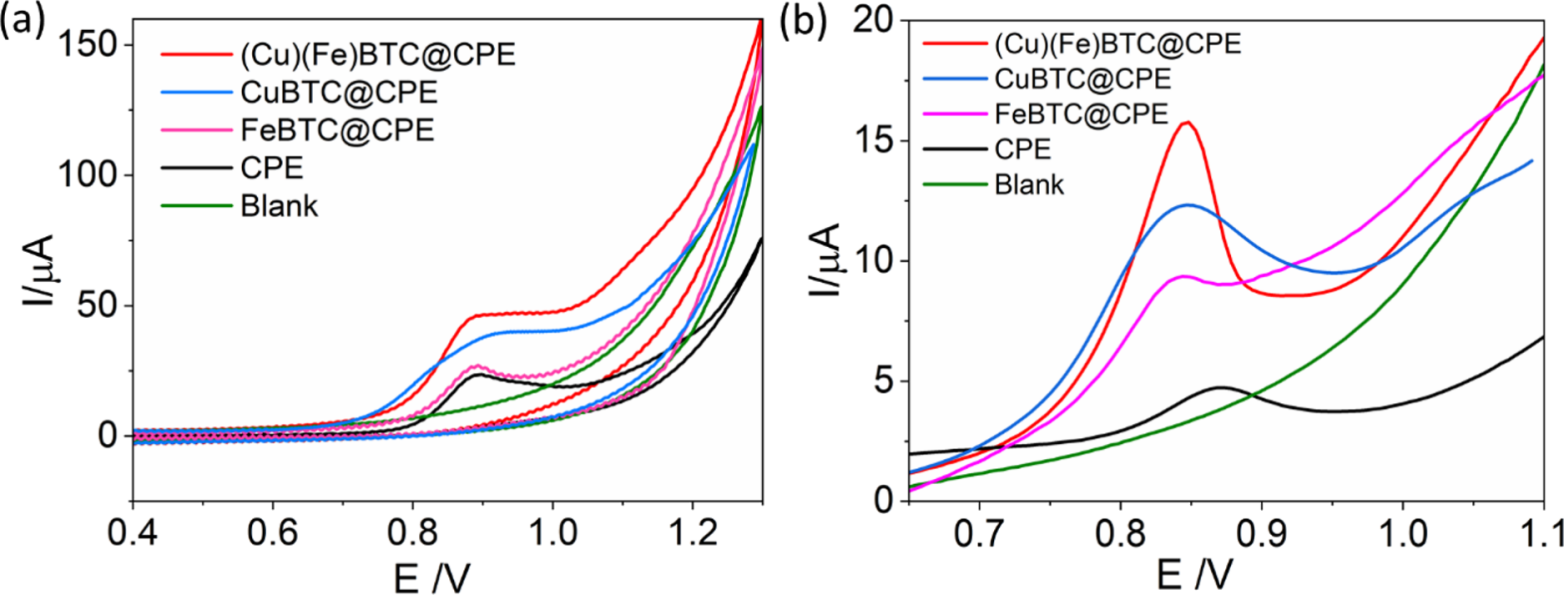
(a) Cyclic voltammograms of (Cu)(Fe)BTC@CPE and CPE electrodes in a 200 µM ENR solution in PBS at scan rate of 0.1 V/s The green line shows a voltammogram measured in ENR-free solution. (b) Square wave voltammograms of the studied electrodes in 1 μM ENR solution at an accumulation time of 90 s. The green line shows a voltammogram measured in ENR-free solution.

The electrochemical behaviour of ENR was studied on both CPE and modified electrodes using cyclic voltammetry in the potential window from 0.4 to 1.2 V. A modified electrode containing 5% CuBTC and 5% FeBTC was used for the investigation. The green line in Figure 6a shows the voltammogram in ENR-free blank solution with no electrochemical response for ENR. In phosphate-buffered saline (PBS) containing ENR, there is a peak at 0.85 V on all four electrodes, attributed to the oxidation of ENR during the anodic scanning. No reduction peak was observed, revealing that the electrochemical reaction mechanism of ENR is irreversible. The experimental results are consistent with previous studies [16,43–44]. Besides, the ENR peak obtained on (Cu)(Fe)BTC was much higher than those on CPE, CuBTC@CPE, and FeBTC@CPE, likely because of the catalytic properties of two MOF materials in the electrode composition.

For the determination of ENR, square wave adsorptive stripping voltammetry (SW-AdSV) was employed because of its superior performance in comparison to the cyclic voltammetric method. Figure 6b illustrates the square wave voltammograms (SWVs) depicting the electrochemical response of ENR in PBS solution containing 1 µM ENR. These SWVs reveal distinct, well-defined, and sharp peaks corresponding to the electrochemical oxidation of ENR. The peak height recorded on the (Cu)(Fe)BTC-modified electrodes is approximately ten times higher than that on the unmodified electrode, and the peak position shifted to more negative values compared to the CPE. This phenomenon highlights the significantly enhanced catalytic activity of the modified electrode. Additionally, the (Cu)(Fe)BTC@CPE electrode exhibited a significantly sharper, more symmetric, and higher peak than both the CuBTC@CPE and FeBTC@CPE electrodes. These observations support the conclusion regarding the synergistic effects of the catalytic activity of CuBTC and the good conductivity of FeBTC, which together contribute to the enhanced performance of the electrode.

### Effect of MOF content on the enrofloxacin signal

**Figure 7:**
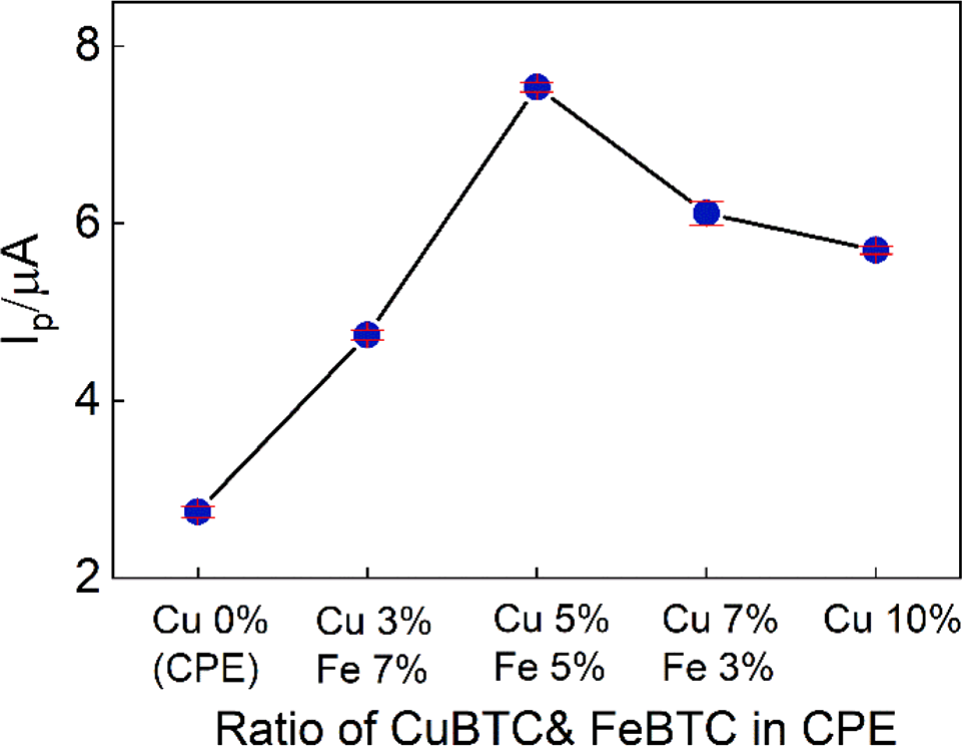
The impact of electrode composition the peak current of 1 µM enrofloxacin.

The electrode has three components, namely, CuBTC, FeBTC, and carbon paste. The carbon paste content was maintained at 90%, resulting in the total of CuBTC and FeBTC being 10%. By altering the proportions of CuBTC and FeBTC in the electrode composition, significant variations in the ENR signal were observed (Figure 7). Specifically, an electrode composition with 0% CuBTC and 10% FeBTC produced an ENR signal of only 1.5 µA. Increasing the CuBTC content to 3% resulted in an ENR signal of 3 µA. The balanced composition of 5% each of CuBTC and FeBTC generated the highest ENR signal of nearly 8 µA among all tested formulations, underscoring the significance of optimizing the ratio of these two components for effective electrochemical detection of ENR. Further increasing the CuBTC content to 7% resulted in a diminished ENR signal of 5 µA. Finally, a composition with 10% CuBTC and 0% FeBTC yielded a peak ENR current of approximately 6 µA. Therefore, the optimal weight percentages of 5% CuBTC and 5% FeBTC were chosen for electrode fabrication in the subsequent experiments.

### Effect of supporting electrolyte on the ENR signal

**Figure 8:**
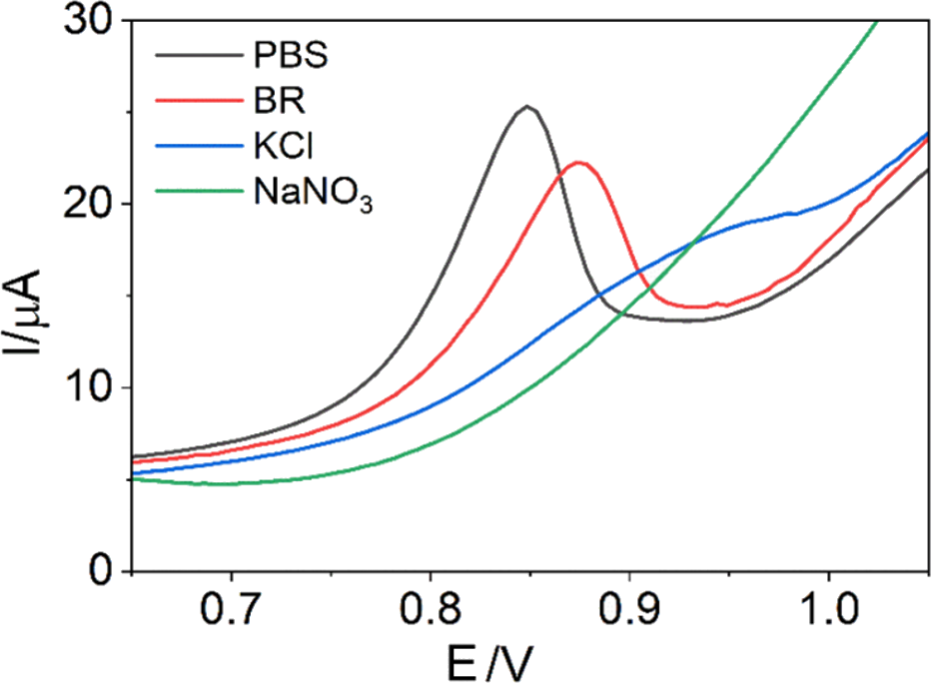
Square wave voltammograms of 1 µM ENR in different electrolytes.

The electrolyte plays a crucial role in the oxidation reaction of ENR. Four electrolytes, that is, PBS, Britton–Robinson (BR), KCl, and NaNO_3_, were tested to determine the most suitable environment for ENR oxidation. The voltammograms in Figure 8 show no clear SWV peak in KCl and NaNO_3_ electrolytes. In contrast, PBS and BR yielded well-defined peaks at 0.85 and 0.88 V, respectively. The ENR peak in PBS is higher, sharper, and more symmetric compared to that in BR. Therefore, PBS was selected as the optimal electrolyte for subsequent experiments.

### Effect of pH of the electrolyte on the ENR signal

**Figure 9:**
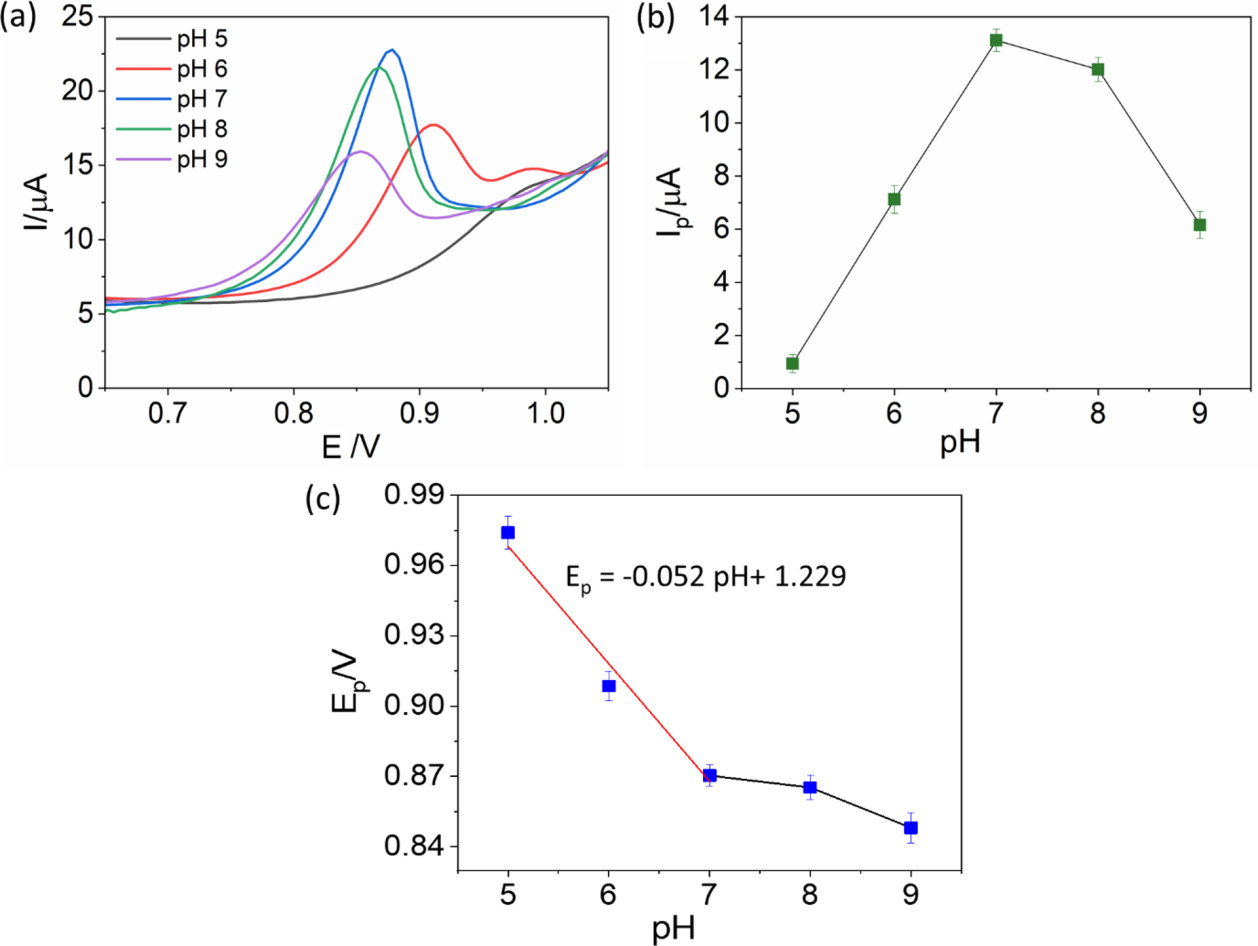
(a) SW-AdSV measurements of (Cu)(Fe)BTC@CPE in 1 µM ENR/PBS solution at different pH values from 6 to 9. (b) Plot of peak current vs pH. (c) Plot of peak potential vs pH.

The effect of pH on the ENR signal was investigated by performing SW-AdSV using the (Cu)(Fe)BTC@CPE electrode in a 1 µM ENR solution in PBS at different pH values (Figure 9a). The height of the ENR peak was significantly affected, as shown in Figure 9b. The ENR peak current increased significantly while the pH changed from 5 to 7 and reached the highest value at pH 7. As the pH value increased further, the peak current gradually decreased to nearly a half at pH 9. Therefore, PBS solution with pH 7 was selected for the experiments in the next section.

The Nernst equation describes the relationship between the peak potential *E*_p_ and pH as follows: *E*_p_ (V) = 0.059(*m*/*n*) pH + *b*, where *n* and *m* denote the quantities of electrons and protons involved in the electrochemical reaction, and *b* is the intercept. Figure 9c shows that, in the pH range from 5 to 7, there is a linear relationship between *E*_p_ and pH value described by the equation *E*_p_ = −0.052 pH + 1.229 with a correlation coefficient of 0.952. The slope of the curve of 0.052 (close to 0.059) indicates that the ratio of protons to electrons participating in the oxidation reaction is 1 to 1, which is in accordance with previous publications [44–45].

### Adsorption time

**Figure 10:**
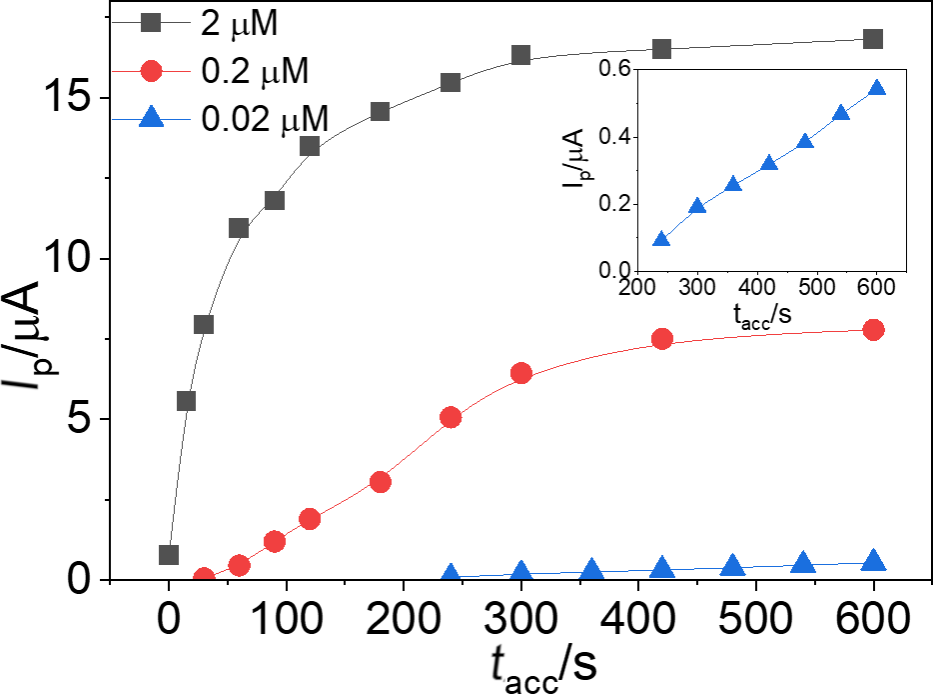
The relationship between ENR peak currents and accumulation time at different concentrations of ENR of 0.02, 0.2, and 2 µM. Inset: rescaled plot for 0.02 µM ENR.

The accumulation time (*t*_acc_) of enrofloxacin on the electrode surface is a critical factor in optimizing the ENR signal. In this study, ENR concentrations ranging from 0.02 to 2 µM were studied with adsorption times varying from 0 to 600 s, as illustrated in Figure 10. At the lowest concentration of 0.02 µM, no peak was observed for *t*_acc_ shorter than 240 s. However, from 240 to 600 s, the peak height increased linearly with the adsorption duration. This indicates that sufficient time is necessary for the ENR molecules to adsorb onto the electrode surface at this low concentration, and the signal improves with longer accumulation times within this range.

For a concentration of 0.2 µM, the peak current increased gradually and reached a steady state at *t*_acc_ exceeding 300 s. This suggests that, at this intermediate concentration, the electrode surface reaches saturation after 300 s of accumulation, beyond which the peak current stabilizes. At the highest concentration tested (2 µM), the peak current experienced a significant increase with increasing *t*_acc_ up to 120 s, followed by a gradual rise, until it reached equilibrium at *t*_acc_ from 300 to 600 s. This behaviour indicates a rapid initial adsorption at high ENR concentration, with the surface reaching almost saturation relatively quickly, and then a slower adsorption to full saturation. These observations highlight the importance of optimizing *t*_acc_ for different ENR concentrations to achieve the best analytical signal. Longer accumulation times are beneficial at lower concentrations, while higher concentrations require shorter times to reach equilibrium.

### Repeatability and reproducibility of the (Cu)(Fe)BTC@CPE electrode

**Figure 11:**
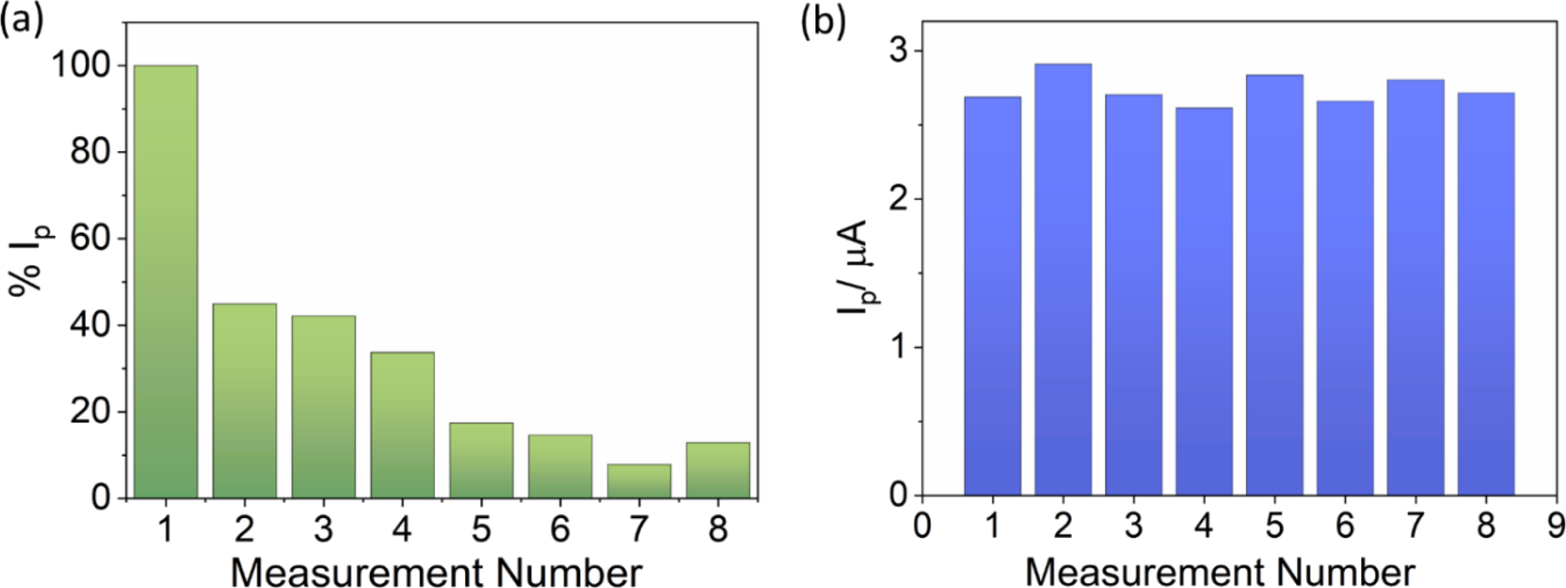
SW-AdSV measurements of successive measurements obtained in 0.20 µM ENR solution using electrodes (a) without renewal of the electrode surface and (b) with renewal of the electrode surface.

Figure 11a depicts eight successive measurements conducted in 0.20 µM ENR solution using a single electrode without renewing the electrode surface. Clearly, in the second measurement, the ENR response decreased dramatically by over 50%. In the seventh and eighth measurements, the peak current of ENR remained at only about 15%. This phenomenon arises from the reaction product covering the active working area of the electrode, resulting in the low peak current observed in these measurements.

The electrode reproducibility performance was evaluated by comparing SWV signals in 0.2 µM ENR solution obtained on an electrode whose surface was renewed before each measurement (Figure 11b). The data indicated slight fluctuations in peak currents among eight measurements, with a relative standard deviation (RSD) of 3.83%. These results demonstrate good reproducibility of the developed electrodes.

### Calibration curve

**Figure 12:**
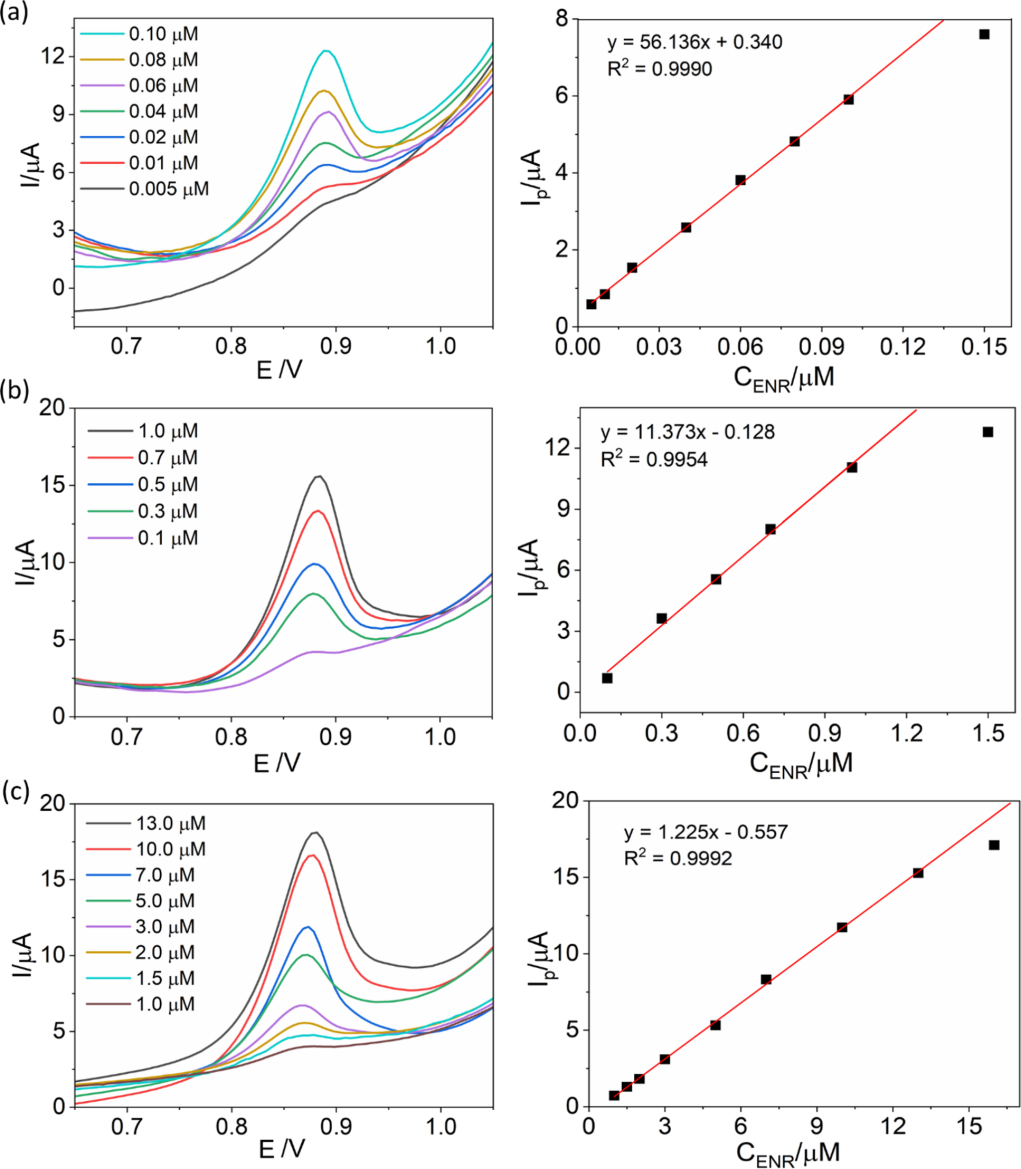
SW-AdSV measurements of ENR and calibration curves for (a) 0.005–0.100 µM (*t*_acc_ of 600 s), (b) 0.1–1.0 µM (*t*_acc_ of 90 s), and (c) 1–13 µM (*t*_acc_ of 0 s).

Under the above optimal conditions, a calibration curve was established in the ENR concentration range from 0.005 to 0.100 µM at *t*_acc_ of 600 s (Figure 12a). Increasing SWV peaks as a function of the concentration were observed, and a linear relationship was established, described by the regression equation *I*_p_ (µA) = 56.136 × *C*_ENR_ (µM) + 0.340. SW-AdSV measurements were carried out with ENR concentrations from 0.1 to 1.0 µM to build the calibration curve at *t*_acc_ of 90 s. The obtained signals, as shown in Figure 12b, exhibit a linear correlation between the ENR oxidation peak height and ENR concentration, described by the regression equation *I*_p_ (µA) = 11.373 × *C*_ENR_ (µM) + 0.128 (*R*^2^ = 0.9954). Figure 12c illustrates the calibration curve established for ENR in the concentration range from 1 to 13 µM at an accumulation time of 0 s. The linear relationship obtained is represented by the regression equation *I*_p_ (µA) = 1.225 × *C*_ENR_ (µM) + 0.557 (*R*^2^ = 0.9992). The consistently high correlation coefficients indicate the robust performance of the developed sensor.

From the calibration curve at the lowest concentration range from 0.005 to 0.100 µM, a detection limit for ENR of 3 nM was calculated through the formula LOD = 3σ/*b*, where σ is the standard deviation of blank signals, and *b* is the slope of the regression line. The LOD is quite comparable to those in previous studies [16,46–49]. Additionally, the sensitivity of the sensor was calculated to be 56.14 µA/µM.

### Anti-interference study

**Figure 13:**
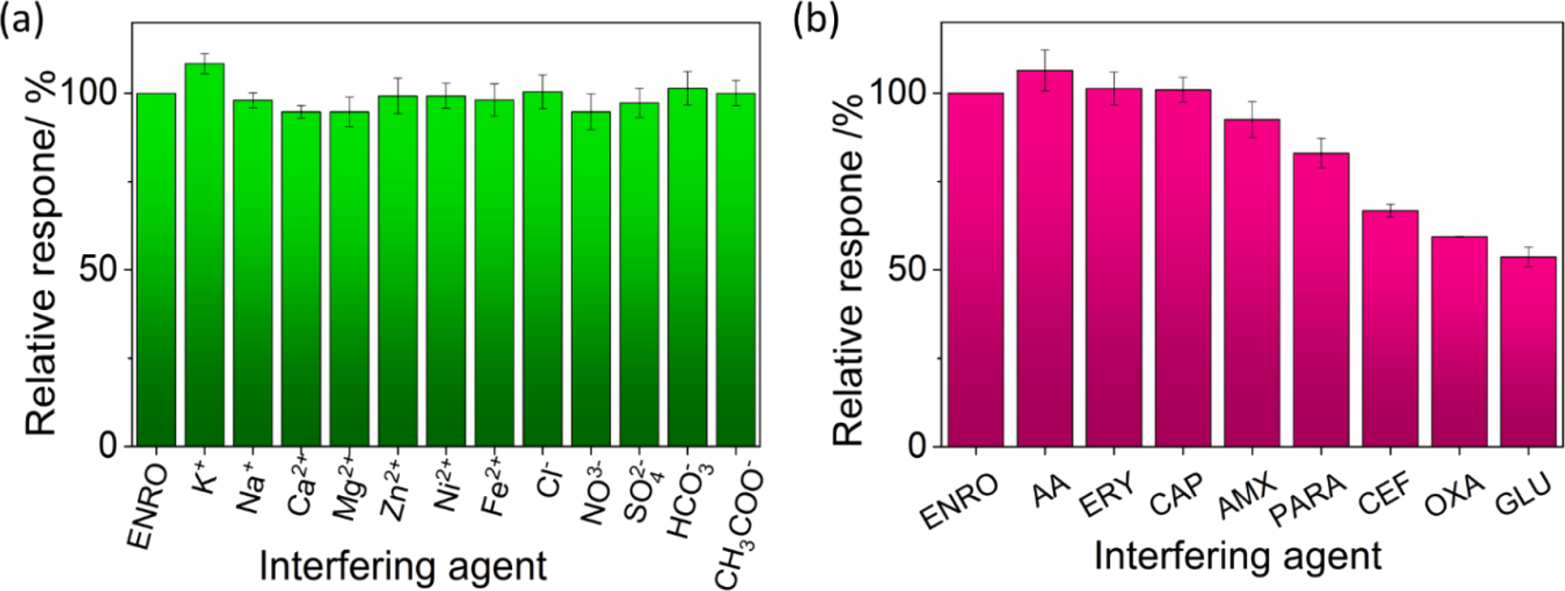
Influence of (a) inorganic ions and (b) organic compounds on the ENR signal.

This study aimed to investigate the influence of organic and inorganic substances on the performance of a sensor by evaluating the changes in the ENR signal when these substances were present in the analytical solution. Figure 13 illustrates that, at a concentration 100 times higher than that of ENR, ions including K^+^, Na^+^, Ca^2+^, Mg^2+^, Zn^2+^, Ni^2+^, Cl^−^, NO_3_^−^, SO_4_^2−^, HCO_3_^−^, and CH_3_COO^−^ had a negligible impact on the ENR signal, leading to a reduction in the ENR signal of less than 5%. Regarding the influence of organic substances, when their concentration was 10 times higher than that of ENR, the impact on the ENR signal was small for ascorbic acid (AA), erythromycin (ERY), chloramphenicol (CAP), paracetamol (PARA), and amoxicillin (AMX). In contrast, cephalosporin (CEF), oxalic acid (OXA), and glucose (GLU) significantly reduced the signal, bringing it down to approximately 30%. At a 50-fold concentration, CAP and PARA still had no noticeable effect on ENR analysis. However, the presence of AA and CAP substantially affected the ENR signal, decreasing it to around 60% (data not shown here). It is worth noting that, within this concentration range, no ENR signal was detectable in the presence of CEF, OXA, or GLU. The experimental findings indicate that the sensor demonstrated good performance in detecting ENR in samples with a complex matrix.

### Analysis in real sample matrices

The performance of the developed sensor was assessed by quantifying ENR levels in tap water and lake water. Different amounts of ENR were added to samples, ranging from 0.2 to 3.0 µM. ENR concentrations were determined using the standard addition method (Table 1). Notably, no ENR was initially detected in the original samples. However, in the spiked samples, the sensor yielded favourable results with recoveries ranging from 90.2% to 121.3%, indicating the reliability of the sensor and its effectiveness in real-world applications.

**Table 1:** Analysis of ENR in spiked samples conducted with the (Cu)(Fe)BTC@CPE sensor.

Sample	*C*_ENR_ (μM)		Recovery (%)

	added concentration	found concentration	

tap water	0	not detected	—

	0.2	0.229	114.5

	0.4	0.434	108.5

	0.6	0.634	105.6

	1.0	1.108	110.8

	2.0	2.029	101.5

	3.0	3.639	121.3

lake water	0	not detected	—

	0.2	0.181	90.5

	0.4	0.392	98.0

	0.6	0.606	101.0

	1.0	0.923	92.3

	2.0	1.854	92.7

	3.0	2.706	90.2

## Conclusion

CuBTC and FeBTC metal-organic frameworks (MOFs) were successfully synthesized using a solvothermal method. The mixture of these MOFs demonstrated a high surface area, substantial total pore volume, and small capillary diameters. This hybrid material was then utilized as an electrode material for an electrochemical sensor designed to detect enrofloxacin through adsorptive stripping square wave voltammetry. The sensor fabricated with an optimal weight ratio between CuBTC and FeBTC (5:5) exhibited the highest signal for enrofloxacin detection. Notably, the sensor showed excellent performance with good reproducibility (RSD = 3.83%), a large dynamic range from 0.05 to 13 µM, a low detection limit of 3 nM, and high selectivity. Under the experimental conditions, the sensor provided satisfactory recoveries ranging from 90.2% to 121.3% in both tap water and lake water samples.

## Experimental

### Reagents and apparatus

#### Reagents

Enrofloxacin (ENR, C_19_H_22_FN_3_O_3_, 98%) was purchased from Thermo Fisher Scientific. Graphite powder and paraffin oil were supplied by Thermo Scientific Acros. Trimesic acid (H_3_BTC, 98%), CuCl_2_·2H_2_O (≥99%), FeCl_3_·6H_2_O (98%), *N*,*N*-dimethylformamide (DMF, 98%) and C_2_H_5_OH (96%), H_3_PO_4_ (85%), KOH (≥85.0%), KH_2_PO_4_ (≥99.5%), K_2_HPO_4_ (≥98.0%), KCl (>99.5%), and HCl (37%) were bought from Fisher and Sigma-Aldrich. All chemicals and reagents were of analytical grade and used without further purification. K_3_[Fe(CN)_6_] (≥99.5%) and K_4_[Fe(CN)_6_] (≥99.5%) were purchased from China. Phosphate-buffered saline (PBS) was prepared by mixing 0.2 M KH_2_PO_4_ and 0.2 M K_2_HPO_4_ in double-distilled water. The pH of the solution used for the optimization of electrolyte solution was changed from 6.0 to 9.0 by controlling the amount of these phosphate salts. Enrofloxacin stock solution was prepared by dissolving ENR powder in double-distilled water. The ENR solutions with desired concentrations were prepared daily by diluting the ENR stock solution in with PBS solution.

#### Apparatus

The electrochemical properties of the developed electrode and enrofloxacin were evaluated through cyclic voltammetry by using a custom-made potentiogalvanostat (supplied by Laboratory of Informatics in Chemistry, Institute of Chemistry, Vietnam Academy of Science and Technology). Electrochemical impedance spectroscopy (EIS) was performed by using an Autolab PGSTAT 302 in a frequency range of 100 kHz to 0.01 Hz. A three-electrode electrochemical setup was used including a platinum wire counter electrode, an Ag/AgCl/saturated KCl reference electrode, and a carbon paste electrode (CPE) or MOF-modified electrodes as working electrodes.

Physical and chemical properties of MOFs were characterized by using field-emission scanning electron microscopy (FESEM, Hitachi S-4800, Japan), energy-dispersive X-ray spectroscopy (EDX, Horiba 7593-H, England), and X-ray photoelectron spectroscopy (XPS, Thermo VG Multilab 2000).

### Synthesis of CuBTC, FeBTC MOFs

CuBTC and FeBTC were synthesized using the solvothermal method as described in our previous reports [37–38]. Specifically, 12 mmol of trimesic acid was fully dissolved in 120 mL of *N*,*N*-dimethylformamide (DMF) (solution 1), and 12 mmol of the chloride salt of the corresponding metal was dissolved in 40 mL of water (solution 2). Solution 1 was then slowly added to solution 2 under vigorous stirring for 30 min before being transferred to a Teflon bottle. The mixture was heated at 150 °C for 12 h and stirred continuously to facilitate the MOF formation process. Finally, the synthesized powder was separated from the solution using a centrifuge at 6000 rpm, washed several times with DMF and ethanol, and dried to obtain CuBTC and FeBTC MOFs.

### Preparation of (Cu)(Fe)BTC@CPE

Modified electrodes were prepared using a standardized procedure. CuBTC and FeBTC were carefully mixed to obtain a mixture called (Cu)(Fe)BTC. Then, paraffin oil, graphite powder, and (Cu)(Fe)BTC powder were combined in various weight ratios (as specified in Table 2) and thoroughly mixed in an agate mortar. The resulting mixture was then tightly packed into a Teflon electrode body (inner diameter 5 mm) to create the modified electrode named (Cu)(Fe)BTC@CPE with a geometrical surface area of 19.63 mm^2^. Prior to experimentation, the electrode surface was polished using a balance paper to achieve a smooth surface. Carbon paste-modified electrodes containing either CuBTC or FeBTC were also fabricated for comparison and labelled as CuBTC@CPE and FeBTC@CPE, respectively.

**Table 2:** Components of the studied electrodes.

Electrode	Electrode component (wt. %)		

	CuBTC	FeBTC	Carbon paste

1	0	0	100
2	3	7	90
3	5	5	90
4	7	3	90
5	10	0	90

### Detection of enrofloxacin

Enrofloxacin was electrochemically detected by using square wave adsorptive stripping voltammetry (SW-AdSV) in PBS. Briefly, the modified electrode was immersed in the analytical solution for different accumulation times (0, 90, and 600 s) under stirring for enrofloxacin adsorption on electrode surface. Then, the SW-AdSV response of enrofloxacin was recorded in the potential window of 0.6–1.1 V.

Spiked samples of tap water and lake water were used to evaluate the performance of the modified electrode. After spiking known concentrations of enrofloxacin into the water samples, the added concentration was determined using the standard addition method. All experimental work was performed at room temperature (25 ± 1 °C).

## Data Availability

All data that supports the findings of this study is available in the published article and/or the supporting information of this article.
